# Bioinspired Tuning of Hydrogel Permeability-Rigidity Dependency for 3D Cell Culture

**DOI:** 10.1038/srep08948

**Published:** 2015-03-10

**Authors:** Min Kyung Lee, Max H. Rich, Kwanghyun Baek, Jonghwi Lee, Hyunjoon Kong

**Affiliations:** 1 Department of Chemical and Biomolecular Engineering, Institute of Genomic Biology, University of Illinois at Urbana-Champaign, Urbana, Illinois, 61801, USA; 2 Department of Materials Science and Engineering, University of Illinois at Urbana-Champaign, Urbana, Illinois, 61801, USA; 3 Department of Chemical Engineering and Materials Science, Chung-Ang University, Seoul, 156-756, Korea; 4 Department of Chemical Engineering, Soongsil University, Seoul, Korea

## Abstract

Hydrogels are being extensively used for three-dimensional immobilization and culture of cells in fundamental biological studies, biochemical processes, and clinical treatments. However, it is still a challenge to support viability and regulate phenotypic activities of cells in a structurally stable gel, because the gel becomes less permeable with increasing rigidity. To resolve this challenge, this study demonstrates a unique method to enhance the permeability of a cell-laden hydrogel while avoiding a significant change in rigidity of the gel. Inspired by the grooved skin textures of marine organisms, a hydrogel is assembled to present computationally optimized micro-sized grooves on the surface. Separately, a gel is engineered to preset aligned microchannels similar to a plant's vascular bundles through a uniaxial freeze-drying process. The resulting gel displays significantly increased water diffusivity with reduced changes of gel stiffness, exclusively when the microgrooves and microchannels are aligned together. No significant enhancement of rehydration is achieved when the microgrooves and microchannels are not aligned. Such material design greatly enhances viability and neural differentiation of stem cells and 3D neural network formation within the gel.

Design of hydrogels used for 3D cell immobilization and culture has focused mainly on tailoring permeability, which significantly affects molecular transport and subsequent viability and phenotypic activities of cells^1^,^2^,^3^,^4^. It is common to modulate the gel permeability by controlling the number of cross-links and/or introducing micropores^5^,^6^,^7^. For example, a hydrogel formulated to have a smaller number of cross-linking junctions presents larger pores, and, subsequently, an increased molecular diffusivity in the matrix. Separately, micropores introduced into a hydrogel contribute to increasing permeability of the gel; however, uncontrollable numbers of non-passing and isolated pores limit the range of control over permeability^6^. More importantly, these traditional approaches dramatically decrease rigidity and negatively influence the structural integrity of a hydrogel, specifically in a condition subject to external mechanical forces^8^.

Interestingly, several marine organisms present unique microstructure to facilitate molecular transports through their elastic tissue. For instance, the skin of blue whales and brain corals exhibit grooved textures so that the skin can uptake a larger mass of molecules including water when compared to flat-surfaced skin^9^. Separately, most plants present anisotropically aligned vascular bundles throughout their stems, so water and nutrients taken up by the root are supplied to all parts of the plant body^10^. Inspired by these natural systems, we hypothesized that a hydrogel engineered to present microgrooves on its surface would significantly elevate gel permeability without appreciably changing the mechanical properties of the gel (Figure 1).

Additionally, incorporation of microchannels into the microgrooved gel would further improve mass transports through the gel, specifically when microchannels are aligned with microgrooves on the surface. We examined this hypothesis by introducing microgrooves on an alginate gel surface using a microstamping technique. A diffusion-based computational simulation was used to guide the geometry of microgrooves. The microgrooved hydrogel was also uniaxially frozen, and lyophilized in order to create microchannels. Microgrooves and microchannels were aligned in parallel with or orthogonal to the microgrooves to systematically evaluate effects of alignments^11^. Permeability of a resulting hydrogel was evaluated by measuring water diffusivity in the gel using magnetic resonance imaging (MRI) system. Multipotent, bone marrow-derived stem cells (BMSCs) were cultured in these hydrogels to evaluate the effect of gel permeability on viability and neural differentiation of stem cells. Taken together, the results of this study will greatly serve to improve controllability of transport and mechanical properties of a wide array of materials used for cell-based bioscience and bioengineering studies.

## Results and Discussion

### Simulation-guided Assembly of microgrooved hydrogels

First, a 2D finite element method-based computational simulation was conducted to understand and optimize the geometry of microgrooves that can significantly enhance molecular diffusion into a gel (Figure 2A). Assuming that molecular transport into a gel was solely controlled by diffusion, oxygen concentration was determined by simulating both the oxygen diffusion rate into a gel and the oxygen consumption rate^12^,^13^,^14^ by cells loaded in a hydrogel using Eq. (1).
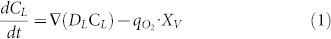
where *C*_L_ is the concentration of oxygen dissolved in the media, *t* is time, *D*_L_ is the diffusivity of oxygen in a media, 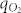 is the oxygen consumption rate per cell, and *X*_v_ is the cell density. The grooves with a depth of 500 μm were simulated while varying the spacing of grooves from 250 to 500 and 1,000 μm. The simulation exhibited that oxygen concentration is dependent on the spacing between microgrooves on the gel surface. Specifically, oxygen concentration in the gel with grooves smaller than 500 μm-spacing would be almost 2.5-fold higher than the gel with flat surface (Figure 2A & 2B). However, decreasing spacing from 500 to 250 μm would make minimal change of oxygen concentration. In addition, a hydrogel with grooves of 1,000 μm-spacing would display a local, limited increase of oxygen concentration around edges of microgrooves. Such simple but important role of surface topology on molecular transports into a gel matrix was not proposed in the past.

To validate the computational simulation, a hydrogel was prepared through a cross-linking reaction of alginate solution under a PDMS stamp with controlled micro-patterns (Figure 3A). The cross-linking reaction between carboxylate groups of alginate and adipic acid dihyrazide (AAD) under the PDMS stamp created hydrogels with pre-defined multiple, microgrooves (Figure 3B). Hydrogels with grooves of spacing at 1,000 and 500 μm were separately prepared, since further reduction of spacing between grooves from 500 to 250 μm would not make any increase of oxygen concentration in the gel. As expected, this topological control of the gel surface minimally changed the bulk elastic modulus of the gel (Figure S1).

Effects of microgroove spacing on water transport into the hydrogel were examined by monitoring time-based diffusion of water originally placed on the gel surface using the MRI (Figure 3C and S2). Similar to the computational simulation of oxygen transport, microgrooved hydrogels displayed faster increases of water bound to a gel matrix than a hydrogel with flat surface as displayed with a more rapid increase of red color level (Figure 3C). Note that mass of water molecules bound to a gel matrix is proportional to the intensity of red color. More interestingly, decreasing the spacing between grooves from 1,000 to 500 μm led to a saturation of the gel with red-colorated bound water within 10 minutes. The gel with grooves of 1,000 μm-spacing displayed localized increase of bound water around microgrooves, similar to computational simulation of oxygen diffusion.

The diffusivity of water into hydrogels (*D*_water_) was quantified by fitting the time-dependent increase of the number of pixels positively colorated at an intensity level higher than 50 to a first-order kinetics, as described by Eq. (2).
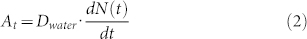
where *A*_t_ is the area of high water concentration at time *t*, *D*_water_ is the diffusivity of water, *N*(t) is the number of positively colorated pixel, and *t* is the time. According to Eq. (2), decreasing spacing of grooves to 500 μm led to a 1.6-fold increase of *D*_water_ attained with the flat hydrogel (Figure S3 and 3D). This dependency of *D*_water_ on the microgroove spacing was attributed to an increase in the surface area-to-volume ratio of the gel (Figure 3D). Total surface area of the hydrogel with grooves of 500 μm-spacing was 1.5 times higher than that of the flat gel.

### Tailoring the hydrogel permeability by aligning microchannels

**Figure 1:**
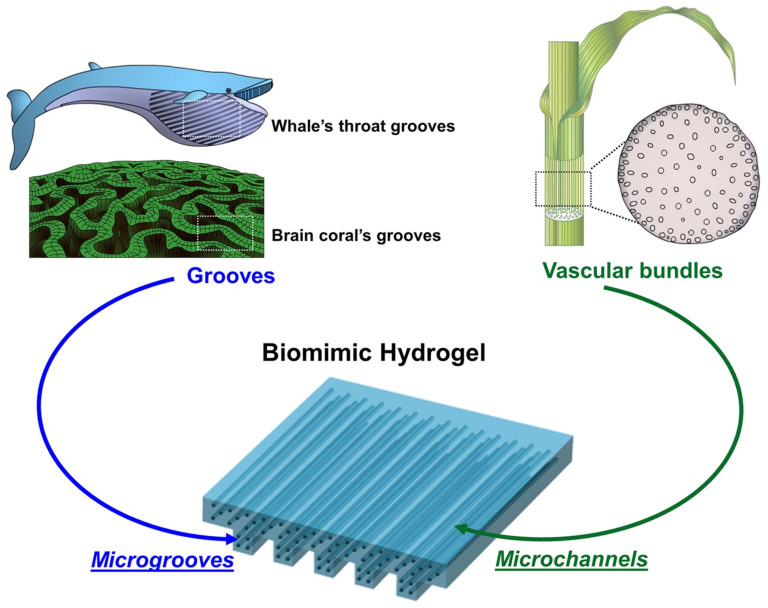
Schematic describing a strategy to control hydrogel permeability by recapitulating microgrooves of marine organisms (e.g., blue whale and brain coral) and microvascular bundles in a stem of plants. The scheme was drawn by Min Kyung Lee.

**Figure 2:**
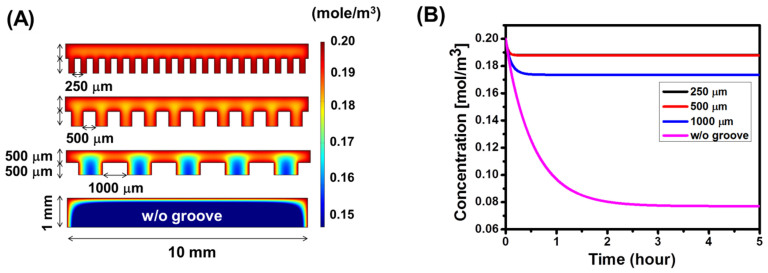
Computational simulation of oxygen distribution in a hydrogel. (A) Two-dimensional distribution of oxygen within cell-encapsulated hydrogels with grooves of different spacing at 250 (top), 500 (middle), and 1,000 μm (bottom). The gel immersed in cell culture media over five hours was simulated. A hydrogel with 1 cm-diameter and 1 mm-thickness was presumed to encapsulate 200,000 cells. The scale bar on the right side represents the oxygen concentration (mol/m^3^). (B) Estimated change of a net oxygen concentration in a hydrogel over time balanced by the diffusion into the gel and the consumption by encapsulated cells.

**Figure 3:**
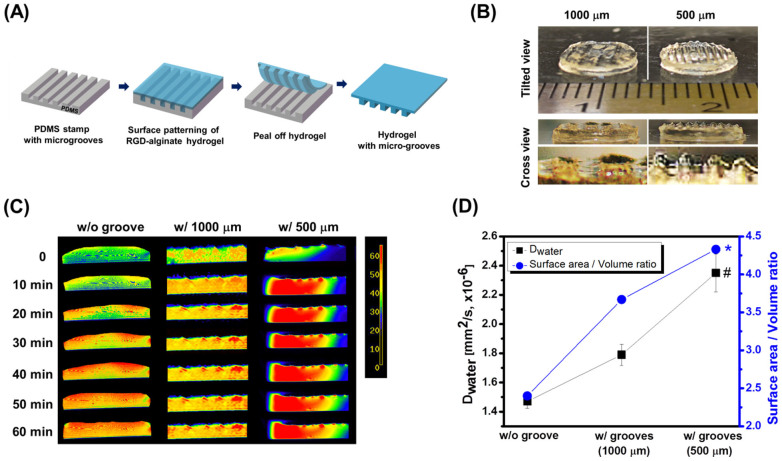
Computational simulation-guided assembly of microgrooved hydrogels. (A) The procedure to prepare the microgrooved hydrogel via *in situ* cross-linking reaction of alginate under a PDMS stamp. (B) Digital images of hydrogels with grooves of controlled spacing at 500 and 1,000 μm. (C) MR images of time-based water distribution in hydrogels. At time zero, 50 μl water was placed on top of the gel surface. The images were developed by averaging 2D image of multiple gel slices with width of 0.5 mm, along the axial plane. (D) Quantified analysis of the diffusivity of water (*D*_water_) in (left axis) and the surface area-to-volume ratio of (right axis) the hydrogels with controlled microgroove spacing. Data are mean ± SE, n = 5/condition (*^,#^*p* < 0.05).

**Figure 4:**
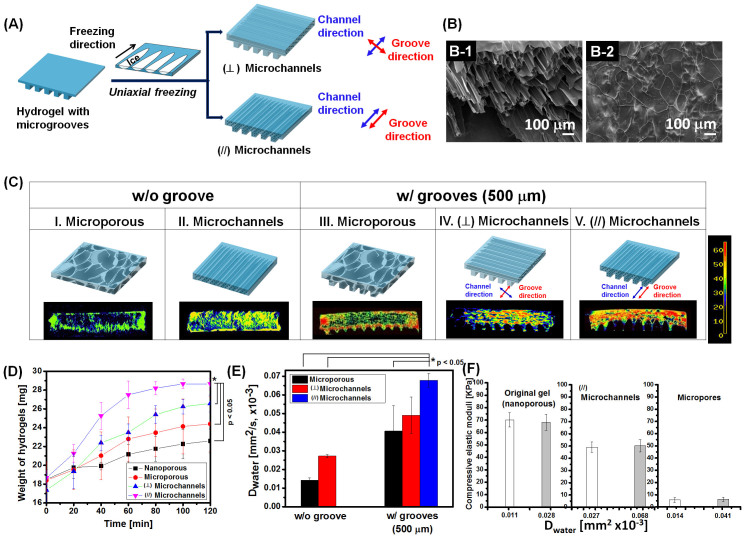
Tailoring the hydrogel permeability by introducing randomly oriented micropores or uniaxial microchannels. (A) Schematic describing the uniaxial freeze-drying process to introduce microchannels aligned perpendicular to microgrooves (upper process) or those aligned with microgrooves (lower process). (B) SEM images of the fractured surface of the microchanneled cryogel (B-1) and microporous cryogel (B-2). (C) MR images that display water distribution in rehydrated, microporous gel and microchanneled gels. The freeze-dried alginate cryogel was rehydrated with deionized water. “W/O groove” represents the hydrogels without microgrooves, and “w/grooves (500 μm)” represents the hydrogels with grooves of 500 μm-spacing. The “(⊥) microchannels” represents the hydrogel in which microchannels were aligned perpendicular to microgrooves on the surface, and the “(//) microchannels” represents the hydrogel in which microchannels were aligned in parallel with microgrooves on the surface. (D) The mass of hydrogels increased over time due to water diffusion into the hydrogel. 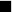 represents an original, nanoporous hydrogel which does not present either micropores or microchannels. 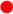 represents a microporous hydrogel prepared by the isotropic freezing process. 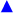 represents the (⊥) microchanneled and 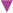 represents the (//) microchanneled hydrogel (*p < 0.05). (E) *D*_water_ of the microporous hydrogel (black bar), (⊥) microchanneled hydrogel (red bar), and (//) microchanneled hydrogel (blue bar) (*p < 0.05). (F) Effect of permeability on compressive elastic modulus of the hydrogels. For each condition, the left bar (i.e., opened one) represents the hydrogel W/O groove and the right bar (i.e., filled one) represents the hydrogel w/grooves (500 μm).

Next, microgrooved hydrogels were modified to present microchannels similar to the microvascular bundle of a plant. In this process, a hydrogel was placed on a copper substrate cooled to sub-zero temperature. Then, multiple ice crystals grew along the direction perpendicular to the copper substrate (Figure 4A). This uniaxial freezing process induced the anisotropic ice crystal growth, while excluding cross-linked polymer chains from the ice crystals. The following lyophilization removed ice crystals, thus resulting in a cryogel with anisotropically aligned microchannels without isolated pores. Depending on the orientation between the gel and the copper substrate, microchannels were aligned either in parallel with microgrooves or perpendicular to the microgroove direction (Figure 4A). The diameter of individual microchannels was 100 ± 15 μm, as confirmed by imaging the fractured surface of the gel with a scanning electron microscope (Figure 4B-1). In contrast, lyophilization of hydrogels frozen in a freezing container that presented sub-zero temperature on all sides produced a cryogel that had randomly oriented micropores with poor opening and interconnectivity, termed as a microporous gel (Figure 4B-2).

In addition, we examined whether the alignment of microchannels with microgrooves of a hydrogel would affect the diffusivity of water. When microgrooved cryogels were re-hydrated by placing water on their surfaces, the cryogel with microchannels displayed a substantial increase in the fraction of bound water (i.e., red colorated area) compared with the microporous gel, as visualized with MR images (Figure 4C and S4). Note that MR images were developed from relaxation rates of water molecules in a gel matrix. The bound water is characterized by lower water relaxation rate than free water, due to a higher binding affinity of water molecules to a gel. Interestingly, the degree of increase in the fraction of bound water was more significant with the gel in which the microchannels were aligned parallel with microgrooves, termed the (//) microchanneled gel. In contrast, the gel in which the microchannels were aligned perpendicular to microgrooves, termed the (⊥) microchanneled gel, displayed a smaller increase of the fraction of bound water than the microporous gel. The role of microchannels in enhancing water transport during rehydration became insignificant with the cryogel without microgrooves.

Additionally, water diffusion into the hydrogel was evaluated by measuring mass of the hydrogel over time while incubating the gel in aqueous media for 2 hrs. Agreed with MR images, the (//) microchanneled gel displayed the most rapid increase of the gel mass (Figure 4D). The diffusivity of water (*D*_water_) was calculated by fitting the increase of gel mass over time to Eq. (3)
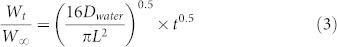
where *W*_t_ and *W*_∞_ are the mass of a hydrogel at time *t* and at equilibrium swelling, respectively, and *L* is the initial gel thickness. Very interestingly, *D*_water_ of the (//) microchanneled gel was 1.8-fold higher than the microporous gel (Figure 4E). In contrast, there was minimal difference of *D*_water_ between the microporous gel and the (⊥) microchanneled gel. Such enhanced water transport within the (//) microchanneled hydrogel is due to the larger number density of interconnected microchannels than the (⊥) microchanneled hydrogel, because of anisotropic growth of ice crystals in parallel with pre-defined microgrooves. The uniaxial freezing process to prepare the (⊥) microchanneled hydrogel likely encountered the discontinued ice crystal growth in the microgrooved area of the gel, because of blocked heat transfer in the microgrooves. Additionally, it is likely that water entry into the (⊥) microchanneled gel is highly limited at the intersection of microgrooves and microchannels.

Whereas the incorporation of microchannels into the hydrogel made a higher increase in the gel permeability than the microporous gel, it made much a smaller decrease of the elastic modulus of the matrix (Figure 4F). Note that the elastic modulus of the (⊥) microchanneled gel was only 1.4-fold smaller than that of the original hydrogel before freeze-drying. This smaller decrease in gel stiffness is likely due to the anisotropically extruded orientation of polymers during uniaxial freezing^15^,^16^, thus compensating effects of microchannels on decreasing stiffness of the gel. In contrast, the elastic modulus of the microporous gel was 14-fold lower than the original gel.

### Analysis of the gel capability to improve metabolic activity and neural differentiation

**Figure 5:**
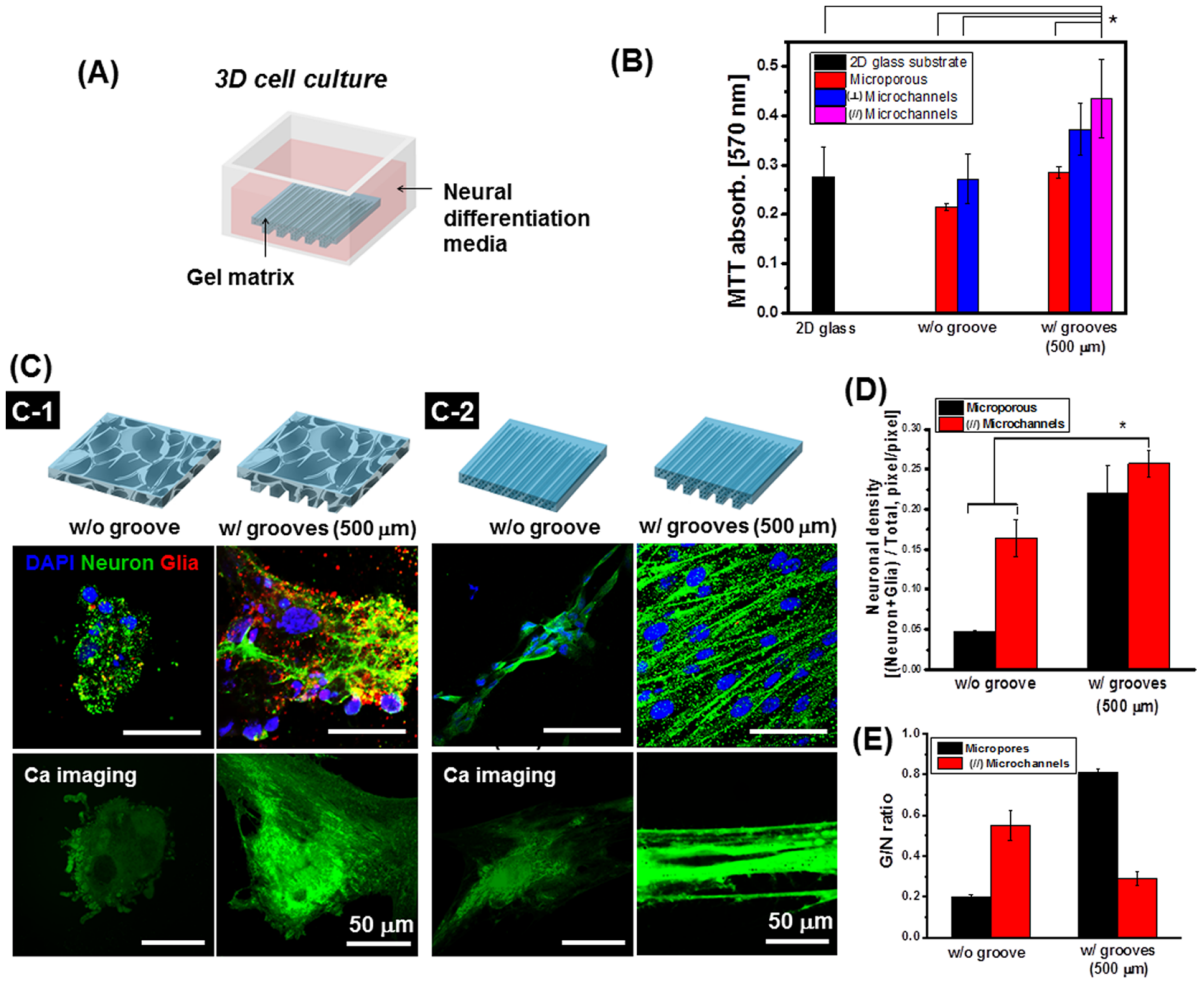
Effects of hydrogel permeability on metabolic activity and neural differentiation of BMSCs. (A) Schematic representation of the 3D BMSC culture using RGD-alginate matrix with controlled permeability. (B) The combined effect of microgrooves and micropores/microchannels on cellular metabolic activity. Data are mean ± SE, n = 5/condition (*p < 0.05). (C) Confocal images of microtubule-associated protein 2 (MAP2, green color) and glial fibrillary acidic protein (GFAP, red color) of neural networks formed within the RGD-alginate hydrogel (2^nd^ row) and Ca^2+^ signal (green color) of differentiated neurons (3^rd^ row). Cells displayed in confocal images represent those located at depth of 60 μm fro the top surface of the gel. At least 600 cells were analyzed from 5 gels per condition. (C-1) Differentiated neuronal cells in the microporous RGD-alginate hydrogels without grooves (i.e., w/o groove) and those with grooves of 500 μm-spacing. (C-2) Differentiated neuronal cells in the (//) microchanneled RGD-alginate hydrogel without microgrooves (i.e., w/o groove) and those with microgrooves of 500 μm-spacing (i.e., w groove). (D) Quantitative analysis of the neuronal density within the RGD-alginate hydrogel: The neuronal density was defined as the areal fraction of glial cells and neuron in the confocal images. (E) Analysis of the ratio between glia and neuron (G/N ratio) in the hydrogels with controlled microgroove spacing. Data are mean ± SE, n = 5/condition (*p < 0.05).

Finally, the microgrooved gels assembled above were modified with cell adhesion oligopeptides^17^ to test whether the controlled hydrogel permeability improves metabolic activity and neural differentiation of bone marrow-derived stem cells (BMSCs) cultured in the gel (Figure 5A). BMSCs were loaded into the hydrogel by rehydrating the freeze-dried cryogel with cell suspension. The elastic modulus of the microchanneled hydrogel used in this cell culture study was 49 ± 4.3 kPa, while that of the microporous gel was 8 ± 1.7 kPa.

According to a MTT assay used to identify positively colored metabolically active cells, BMSCs cultured in the microchanneled hydrogel remained more metabolically active when grooves with 500 μm-spacing were present on the surface (Figure 5B). In addition, the (//) microchanneled hydrogel was more advantageous to increasing the number of metabolically active BMSCs than the (⊥) microchanneled gel. In contrast, the microgrooves on the microporous gel surface did not make a significant contribution to elevating metabolic activity of cells. The MTT absorbance (at 570 nm) of cells cultured on a 2D glass substrate was 0.286 (±0.06), which is almost same as the microporous gel with microgrooves.

Additionally, microgrooves and microchannels introduced into the gel orchestrated to elevate the neural differentiation level of BMSCs evaluated with density of neurons, a ratio of neuron to glial cells, and calcium transport of differentiated cells. Incubation of the BMSC-laden hydrogel in the neural differentiation media activated differentiation of BMSCs to neuronal cells that express microtubule-associated protein (MAP2). BMSCs can also differentiate to glial cells that express glial fibrillary acidic protein (GFAP)^18^,^19^,^20^,^21^. Interestingly, cells cultured in the microporous hydrogel with an elastic modulus of 10 kPa formed a cluster independent of microgrooves on the gel surface, while those cultured in the microchanneled hydrogel with an elastic modulus of 50 kPa were fully stretched (Figure 5C). The cellular clusters formed in the soft microporous hydrogel were morphologically similar to cells loaded into a microchanneled hydrogel with an elastic modulus of 7 kPa (results not shown). It is also noteworthy that number of cells in the gel was larger with presence of microgrooves for both microporous and microchanneled hydrogels.

More interestingly, compared with cells cultured in the microporous gel (see second row in Figure 5C-1), BMSCs differentiated within the (//) microchanneled hydrogel formed a larger density of neurons and a smaller number of glial cells (see second row in Figure 5C-2 & 5D). Interestingly, differentiated neuronal cells aligned to each other in the (//) microchanneled gel, while differentiated ones in the microporous gel were aggregated. More interestingly, the glial to neuron ratio with the (//) microchanneled gel was 4-fold smaller than the microporous gel (Figure 5E).

The microgrooves of the gel played an important role on the neural differentiation level and the glial-to-neuron ratio, termed G/N ratio. For this analysis, the microchanneled hydrogel free of microgrooves was used as a control. According to the water diffusion analysis presented in Figure 4E, diffusivity of water in the control gel was two-fold smaller than the (//) microchanneled gel. Interestingly, neural density in the control gel system was almost two-fold lower than that attained with the (//) microchanneled gel. Conversely, the G/N ratio was two-fold higher with the control gel, which implicates that the (//) microchanneled gel with a higher permeability greatly supported differentiation of BMSCs to neuron-like cells.

We further analyzed the action potential morphology of differentiated neuron-like cells by imaging calcium channels of the cell. The level of calcium signals in the differentiated neuronal cells was evaluated with fluorescence arising from Fluo-4 AM bound with calcium ions in living cells. No significant difference in the level of calcium ion was detected between undifferentiated BMSCs and differentiated neuronal cells, when they were cultured on a 2D substrate (Figure S5). The neuronal cells differentiated in the gel without microgrooves did not present a higher level of calcium ions, either (see the third row in Figure 5C, S6 and S7). In contrast, with the microgrooved gel, neuronal cells in the (//) microchanneled gel expressed a 2.5-fold higher level of anisotropically aligned calcium channels than those in the microporous gel.

According to previous studies conducted by activating neural differentiation of BMSCs on hydrogels with controlled stiffness, neural differentiation of BMSCs is enhanced on a hydrogel with similar softness of brain tissue^22^. In contrast, our finding that neural differentiation of BMSCs cultured in a gel is enhanced with the stiffer (//) microchanneled gel compared to the softer microporous gel implicates that transport property of a matrix is a more pre-dominant factor than mechanical property with the 3D cell culture. However, controlling stiffness of the (//) microchanneled gel may allow us to tune mechanical signals of the matrix at a given permeability and attain a synergy to further elevate neural differentiation levels of BMSCs.

By combining MR images of the rehydrated gel with cellular metabolic and phenotypic activities, the results of this study address the beneficial role of bound water on 3D cell culture and further tissue development. We suggest that water molecules bound to a gel matrix do not only facilitate molecular transports into a gel matrix but also support cellular adhesion to microchannels of a gel matrix. Conversely, free water molecules implicate insufficient rehydration of a gel matrix, which leads to limited molecular transports and cell adhesion.

In addition, it is noteworthy that the difference of cellular metabolic and phenotypic activities between the microporous and microchanneled hydrogel may be partially attributed to the difference of mechanical stiffness. Cellular clusters formed in the microporous gel were morphologically similar to those found in a microchanneled gel with similar softness. Therefore, to better address the underlying mechanism, it is necessary to systematically study balanced effects of matrix permeability and stiffness on cellular response in a 3D matrix in future studies.

## Conclusion

Overall, this study successfully demonstrated that bio-inspired assembly of a hydrogel to present microchannels aligned in parallel with microgrooves of optimal spacing (i.e., 500 μm) allowed us to tune mass transports in the gel, without significantly softening the gel. The perpendicular alignment between microgrooves and microchannels or the incorporation of randomly aligned micropores into the hydrogel with microgrooves did not improve water diffusion into the 3D gel matrix. We suggest that such advanced tuning of the dependency between permeability and stiffness of the gel should be attributed to the orchestrated controls of microgrooves-induced surface area, interconnectivity of microchannels, and organized densification of polymers resulting from uniaxial freezing. Therefore, we could demonstrate the improved cellular metabolic and phenotypic activities in a stiff but permeable gel system. We envision that such bio-inspired orchestration of surface topology and microchannel architecture of a gel would be highly useful to culturing a wide array of stem and progenitor cells for fundamental bioscience studies and cell therapies. It would be also advantageous to improving viability and metabolic activities of yeasts and bacterial cells used in a variety of biochemical processes^23^. Additionally, the material assembly principle can be readily extended to fabricating a variety of material systems used in both biological and non-biological applications.

## Methods

### Computational simulation

The oxygen distribution in a cell-laden gel was simulated using the Comsol Multiphysics 4.3. A 200,000 cell-laden gel disk with 1 cm-diameter and 1 mm–thickness was simulated. The spacing of groove with 500 μm-depth was varied from 200 to 500, and 1,000 μm. The initial concentration of oxygen and the oxygen consumption of each cell were approximated to be 200 μmol/L and 100 fmol/h, respectively^12^,^13^,^14^. The diffusion coefficient of oxygen in hydrogels was approximated to be 2 × 10^−10^ m^2^/s. All simulations were made in meters, seconds, and kilograms. Each sample was simulated for a period of 5 hours.

### Hydrogel assembly

Based on simulation results, alginate hydrogels with microgrooves of controlled spacing were assembled by activating cross-linking reaction between alginate and adipic acid dihydrazide on a poly(dimethoxysiloxane) (PDMS) stamp with pre-defined, positive patterns. Both unmodified alginate and alginate conjugated with oligopeptides containing Arg-Gly-Asp sequence^17^, termed RGD peptides, were used to prepare the gel. The microchanneled hydrogel was prepared by placing the flat or microgrooved gel on a copper plate at sub-zero temperature to induce uniaxially aligned ice crystals followed by lyophilization^11^. The resulting cryogel was rehydrated with DI water or cell suspension. In contrast, the microporous cryogel was prepared by placing the flat or microgrooved gel in a copper chamber at sub-zero temperature followed by lyophilization.

### Hydrogel characterization

The water distribution within the resulting hydrogel was monitored using a 600 MHz Varian Unity/Inova nuclear magnetic resonance (NMR) spectrometer (14.1 T magnet) at room temperature, after dropping the water on the gel surface. The spin echo multi-slice (SEMS) pulse sequence was applied to acquire resonance data, and water density maps were obtained using VNMR 6.1 C software. The repetition time (T_R_) was 500 ms and the echo time (T_E_) was 9.5 ms. Separately, an increase of the gel mass over time was monitored while incubating the cryogel in DI water. In parallel, the gel stiffness was evaluated by measuring a compressive elastic modulus.

### Cell assays

After 24 hours since incorporating BMSCs with a cell number of 5 × 10^4^ into the gel, the number of metabolically active cells within the hydrogels was measured using a MTT cell proliferation assay kit (Invitrogen). Cells plated on a glass with a number of 5 × 10^4^ were also examined as a control. The absorbance of samples treated with MTT reagents following the manufacturer's protocol was measured at 570 nm using a spectrophotometer (Synergy HT, BioTek). The neural differentiation was activated by incubating cells in a neurogenic differentiation media (PromoCell). After 7 days, following fixation and permeabilization, cells were incubated with the rabbit polyclonal anti-microtubule-associated protein 2 (anti-MAP2) (10 μg/mL, Invitrogen) or mouse monoclonal anti-glial fibrillary acidic protein antibody (anti-GFAP) (4 μg/mL, Sigma) overnight. Thereafter, cells were incubated with the secondary fluorescent antibodies to image intracellular MAP2 and GFAP using the laser-scanning confocal microscope (LSM700, Zeiss). Cells located at depth of 60 mm from the top surface of the gel were captured. Calcium channels of the differentiated BMSCs were imaged by staining cells with Fluo-4AM (Invitrogen). The positively stained calcium channel was imaged using the confocal microscope. Approximately 300 cells were imaged in 8 separate gel matrix.

### Statistical analysis

All averaged data are presented as means ± SE. To determine significance, comparisons between groups were performed by one-way ANOVA followed by Tukey's Multiple Comparison Test (p < 0.05).

## Author Contributions

M.K.L., J.L. and H.K. designed research; M.K.L. and M.H.R. performed research; Computational simulation was performed by K.B.; M.K.L. and H.K. wrote the main manuscript text. All authors reviewed the manuscript.

## Supplementary Material

Supplementary Information Supplementary Information

## References

[b1] Drury J. L. & Mooney D. J. Hydrogels for tissue engineering: scaffold design variables and applications. Biomaterials 24, 4337–4351 (2003). 12922147 10.1016/s0142-9612(03)00340-5

[b2] Brandl F., Sommer F. & Goepferich A. Rational Design of hydrogels for tissue engineering: impact of physical factors on cell behavior. Biomaterials 28, 134–146 (2007). 17011028 10.1016/j.biomaterials.2006.09.017

[b3] Jen A. C., Wake M. C. & Mikos A. G. Review: Hydrogels for cell immobilization. Biotechnol. Bioeng. 50, 357–364 (1996). 18626984 10.1002/(SICI)1097-0290(19960520)50:4<357::AID-BIT2>3.0.CO;2-K

[b4] Lee K. Y. & Mooney D. J. Hydrogels for tissue engineering. Chem. Rev. 101, 1869–1879 (2001). 11710233 10.1021/cr000108x

[b5] Hwang C. M. *et al.* A. Fabrication of three-dimensional porous cell-laden hydrogel for tissue engineering. Biofabrication 2, 1–12 (2010). 10.1088/1758-5082/2/3/035003 PMC3282162 20823504

[b6] Stachowiak A. N., Bershteyn A., Tzatzalos E. & Irvine D. J. Bioactive hydrogels with and ordered cellular structure combine interconnected macroporosity and robust mechanical properties. Adv. Mater. 17, 399–403 (2005).

[b7] Park J. H. *et al.* Microporous cell-laden hydrogels for engineered tissue constructs. Biotechnol. Bioeng. 106, 138–148 (2010). 20091766 10.1002/bit.22667 PMC2847036

[b8] Cha C., Kim E.–S., Kim I. W. & Kong H. J. Integrative design of a poly(ethylene glycol)-poly(propylene glycol)-alginate hydrogel to control three dimensional biomineralization. Biomaterials 32, 2695–2703 (2011). 21262532 10.1016/j.biomaterials.2010.12.038

[b9] Lambertsen R. H. Internal mechanism of rorqual feeding. J. Mammal. 64, 76–88 (1983).

[b10] Dimond A. E. Pressure and flow relations in vascular bundles of the tomato plant. Plant Physiol. 41, 119–131 (1966). 16656221 10.1104/pp.41.1.119 PMC1086308

[b11] Lee M. K. *et al.* Glacier moraine formation-mimicking colloidal particle assembly in microchanneled, bioactive hydrogel for guided vascular network construction. Adv. Healthcare Mater. 4, 195–201 (2015). 10.1002/adhm.201400153 24898521

[b12] Van Stroe-Biezen S. A. M., Everaerts F. M., Janssen L. J. J. & Tacken R. A. Diffusion coefficients of oxygen, hydrogen peroxide and glucose in a hydrogel. Anal. Chim. Acta. 273, 553–560 (1993).

[b13] Pattappa G., Heywood H. K., de Bruijn J. D. & Lee D. A. The metabolism of human mesenchymal stem cells during proliferation and differentiation. J. Cell. Physiol. 226, 2562–2570 (2011). 21792913 10.1002/jcp.22605

[b14] Jorjani P. & Ozturk S. S. Effects of cell density and temperature on oxygen consumption rate for different mammalian cell lines. Biotechnol. Bioeng. 64, 349–356 (1999). 10397872 10.1002/(sici)1097-0290(19990805)64:3<349::aid-bit11>3.0.co;2-v

[b15] Lee M. K. & Lee J. A nano-frost array technique to prepare nanoporous PVDF membranes. Nanoscale 6, 8642–8648 (2014). 24865989 10.1039/c4nr00951g

[b16] Lee M. K. & Lee J. Fabrication of ferroelectric polymer nanocrystals with tunable morphologies. Cryst. Growth Des. 13, 671–678 (2013).

[b17] Rowley J. A. & Mooney D. J. Alginate type and RGD density control myoblast phenotype. J. Biomed. Mat. Res. 60, 217–223 (2002). 10.1002/jbm.1287 11857427

[b18] Sanchez-Ramos J. *et al.* Adult bone marrow stromal cells differentiate into neural cells in vitro. Exp. Neurol. 164, 247–256 (2000). 10915564 10.1006/exnr.2000.7389

[b19] Bossolasco P. *et al.* Neuro-glial differentiation of human bone marrow stem cells in vitro. Exp. Neurol. 193, 312–325 (2005). 15869934 10.1016/j.expneurol.2004.12.013

[b20] Kopen G. C., Prockop D. J. & Phinney D. G. Marrow stromal cells migrate throughout forebrain and cerebellum, and they differentiate into astrocytes after injection intoneonatal mouse brains. Proc. Natl. Acad. Sci. USA 96, 10711–10716 (1999). 10485891 10.1073/pnas.96.19.10711 PMC17948

[b21] Hofstetter C. P. *et al.* Marrow stromal cells from guiding strands in the injured spinal cord and promote recovery. Proc. Natl. Acad. Sci. USA 99, 2199–2204 (2002). 11854516 10.1073/pnas.042678299 PMC122342

[b22] Discher D. E., Janmey P. & Wang Y.-L. Tissue cells feel and respond to the stiffness of their substrate. Science 310, 1139–1143 (2005). 16293750 10.1126/science.1116995

[b23] Cha C., Kim S. R., Jin Y.-S. & Kong H. J. Tuning structural durability of yeast-encapsulating alginategel beads with interpenetrating networks for sustained bioethanol production. Biotech. Bioeng. 109, 63–73 (2012). 10.1002/bit.23258 21732329

