# Supplementary material for: Bioinspired Tuning of Hydrogel Permeability-Rigidity Dependency for 3D Cell Culture
Source: Sci Rep. 2015 Mar 10;5:8948. doi: 10.1038/srep08948 (PMC4353999; doi:10.1038/srep08948)
Supplement: Supplementary Information [file srep08948-s1.pdf]

Supporting Information:

Bioinspired Tuning of Hydrogel Permeability-Rigidity Dependency for 3D Cell Culture

Min Kyung Lee[1], Max H. Rich[1], Kwanghyun Baek[2], Jonghwi Lee[3,*], and Hyunjoon Kong[1,*]

[1]Department of Chemical and Biomolecular Engineering, Institute of Genomic Biology, University of Illinois at Urbana-Champaign, Urbana, Illinois, 61801, USA

[2]Department of Materials Science and Engineering, University of Illinois at Urbana-Champaign, Urbana, Illinois, 61801, USA

[3]Department of Chemical Engineering and Materials Science, Chung-Ang University, Seoul, 156-756, Korea

E-mail: jong@cau.ac.kr, hjkong06@illinois.edu

1. Experimental Details

Computational simulation: The oxygen distribution in a cell-laden gel was simulated using the Comsol Multiphysics 4.3. A 200,000 cell-laden gel disk with 1 cm-diameter and 1 mm–thickness was simulated. The spacing of groove with 500 μm-depth was varied from 200 to 500, and 1,000 μm. The initial concentration of oxygen and the oxygen consumption of each cell was approximated to be 200 μmol/L and 100 fmol/h, respectively. The diffusion coefficient of oxygen in hydrogels was approximated to be 2 x 10^{-10} m^2/s. All simulations were made in meters, seconds, and kilograms. Each sample was simulated for a period of 5 hours.

Chemical coupling of RGD peptide to alginate: 1 wt% Alginate (Mw ~ 250,000 g/mol, LF 20/40 from Biopolymer) was dissolved in the (N-morpholino)ethanesulfonic acid (MES) buffer. Then, sulfonated N-hydroxysuccimide (Sulfo-NHS, Thermo Scientific), 1-ethyl-3-(3-dimethylaminopropyl) carbodiimide (EDC, Thermo Scientific) and the oligopeptide with a sequence of GGGGRGDSP, termed RGD peptides (Mimotopes Pty Ltd.), were sequentially added to the alginate solution, and stirred for 1day (molar ratio of uronic acid:sulfo-NHS:EDC:RGD peptide=1:3:2:2). The alginate solution was dialyzed against DI water for 3 days. The water was replaced three times per day. The alginate coupled with RGD peptide (RGD-alginate) was collected through sequential filtration and lyophilization. The RGD-alginate was kept dried in a sterile condition until it is used.

Fabrication of alginate hydrogels with micro-grooves: Based on simulation results, alginate hydrogels with micro-grooves of controlled spacing were assembled by activating cross-linking

reaction between alginate and adipic acid dihydrazide on a poly(dimethoxysiloxane) (PDMS) stamp with pre-defined, positive patterns. Sterile RGD-alginate was dissolved in 0.1 M MES buffer (pH 6.5) at a concentration of 2% (w/v). The RGD-alginate solution was sequentially mixed with sulfo-NHS, adipic acid dihydrazide (AAD, Sigma-Aldrich), and EDC. The molar-ratio of uronic acid:sulfo-NHS:EDC:AAD was kept constant at 1.0:1.0:1.0:0.2. Subsequently, the pre-gel solution was poured onto a poly(dimethoxysiloxane) (PDMS) stamp with pre-defined, positive pattern. Both the height and width of linear posts of the PDMS stamp were kept constant at 500 μm, while varying the spacing of posts from 500 to 1,000 μm. After 10 minutes, hydrogel disks with diameters of 5 mm were punched out using a puncher, and incubated in DI water at room temperature for 12 hrs.

Fabrication of microchanneled or microporous hydrogel: The micro-grooved hydrogel disk was placed on top of a copper plate with thickness of 0.5 mm. Then, the lower part of the copper plate was in contact with a liquid nitrogen bath for 5 minutes, in order to decrease the copper plate temperature near -196 ^{o}C. Depending on the direction to place the gel on the copper substrate, the microchannel was aligned either parallel with micro-grooves or perpendicular to the micro-groove direction. The frozen gel was lyophilized to remove ice crystals and subsequently introduce aligned microchannels.

Separately, the microporous hydrogel was prepared by placing the alginate hydrogel in a copper container at -196 ^{o}C. A diameter of the copper container was designed to be equivalent to a diameter of the hydrogel, in order to make the bottom and wall of the hydrogel disk contact with surface of the copper container. Again, the frozen hydrogel was lyophilized to remove ice from the gel matrix.

Magnetic resonance (MR) imaging of water diffusion into hydrogels: The water distribution within the hydrogel was monitored using a 600 MHz Varian Unity/Inova nuclear magnetic resonance (NMR) spectrometer (14.1 T magnet) at room temperature. The gel disk with 10 mm diameter was inserted into a Radio Frequency coil and DI water droplet (50 μl) was placed on top of the gel. Then, spin echo multi-slice (SEMS) pulse sequence was applied to acquire resonance data and water density maps were obtained using VNMR 6.1 C software. The repetition time (T_R) was 500 ms and the echo time (T_E) was 9.5 ms. Top view images of the gel were taken with the 1.0 x 1.0 cm field of view, 256 x 256 pixel resolution, and each scanned slice was 1 mm thick. The slide view images were taken with the 0.7 x 0.7 cm field of view, 256 x 256 pixel resolution, and each scanned slice was 1 mm thick. The pseudo-colored water density images at each time point were generated by converting the black and white MR image to an 8-bit image in image J (1.46 version, NIH) and subsequently, editing the LUT (Look Up Table) file in order to have a range of colors instead of gray scale.

Measurement of water diffusion into the hydrogel: The freeze-dried microporous or microchanneled cryogels were immersed into deionized water at room temperature. Periodically, the hydrogels were removed from the water, and then excess water was removed from the gel surface with a filter paper. The mass of the hydrogel was recorded over 2 hours.

Measurement of elastic moduli of the hydrogel: Uniaxial compression tests were performed using a mechanical testing system (MTS insight) to measure elastic moduli of the hydrogels before and after lyophilization. Following the incubation in deionized water for 12 hrs, the gel

disks with 1 cm-diameter and 1 mm-thickness were compressed at a rate of 1 mm/min. The elastic modulus (*E*) of the gel was calculated from the linear slope of the stress (σ) versus strain (ε) curve for the first 10% strain.

In vitro cell viability analysis: The number of metabolically active cells within the hydrogels was measured using a MTT Assay kit (Sigma-Aldrich), following the manufacturer's protocol. Briefly, mouse bone marrow stromal cells (BMSCs, ATCC) were introduced into microchannels or micropores of the gel by rehydrating dried matrices with cell suspension. After 24 hours, cells within the hydrogels were incubated in fresh media containing the MTT reagent (0.5 mg/ml) at 37 ^{o}C for 4 hours. After removing the media, 100 μl of DMSO was added per each well. Then, the sample was incubated at 37 ^{o}C for 1 hour, and the absorption was measured at 570 nm using a spectrophotometer (Synergy HT, BioTek).

Neural differentiation of hBMSCs within hydrogels: Human bone marrow stromal cells (hBMSCs) kindly supplied by Lonza were cultured in DMEM supplemented with 10 % FBS. The cells were introduced into microchannels or micropores of the gel by rehydrating the gel with the cell suspension at a cell density of 5x10^{5}/ml. Thereafter, the cell-laden hydrogel was incubated in the neurogenic differentiation media (PromoCell). The media was exchanged with fresh one every day. After 7 days, cells in the hydrogel were fixed with 4% (w/v) paraformaldehyde in PBS and washed three times with PBS supplemented with Ca^{2+} and Mg^{2+}. The cells were permeabilized using 0.3%(w/v) Triton X-100 for 10 min. Then, blocking was performed with PBS dissolved with 5% goat serum overnight. Next, cells in the hydrogel were incubated in PBS dissolved with the rabbit polyclonal anti-microtubule-associated protein 2

(anti-MAP2) (10 μg/mL, Invitrogen) and mouse monoclonal anti-glial fibrillary acidic protein antibody (anti-GFAP) (4 μg/mL, Sigma) overnight. Thereafter, cells were incubated in PBS dissolved with the secondary antibody Alexa Fluor 488 goat anti-mouse (100 μg/mL, Molecular Probes) and Alexa Fluor 568 goat anti-rabbit (10 μg/mL, Molecular Probes) at room temperature for 2 hrs. After staining, samples were washed three times with PBS and incubated in 100 ng/ml DAPI solution for 5 minutes. Finally, intracellular MAP2 and GFAP were imaged using the laser-scanning confocal microscope with 40x oil lens (LSM700, Zeiss). All cells in confocal images were located at depth of 60 μm from top surface of the hydrogel.

Calcium imaging of neural cell differentiated within hydrogels: The hBMSCs differentiated to neuronal cells within the gel were incubated for 60 min at 37 ^{o}C in 5 μM solution of fluo-4 (Bioscience) prepared in EBSS containing 1.8 mM Ca^{2+}. All samples were then washed twice with EBSS and incubated in EBSS for further 15 min at room temperature to allow complete esterification of the stains. Finally, cells stained with fluo-4AM were imaged, and the resulting images were used for analysis of Ca^{2+} signaling. All samples were placed on a sterile coverslip, and mounted on the stage of a confocal microscope (LSM700, Zeiss). A 20x objective lens was used to visualize cells within a field of view of 265 x 265 μm and at a depth of ~50 μm into the gel matrix. Laser excitation light was provided at a wavelength of 488 nm, and fluorescent emissions were collected at wavelengths above 515 nm. For image acquisition, an exposure time of 0.8s was adopted. Approximately 300 cells were imaged in 8 separate gel matrix.

Statistical analysis: All averaged data are presented as means ±SE. To determine significance, comparisons between groups were performed by one-way ANOVA followed by Tukey's Multiple Comparison Test ($p < 0.05$).

2. Supporting Figures

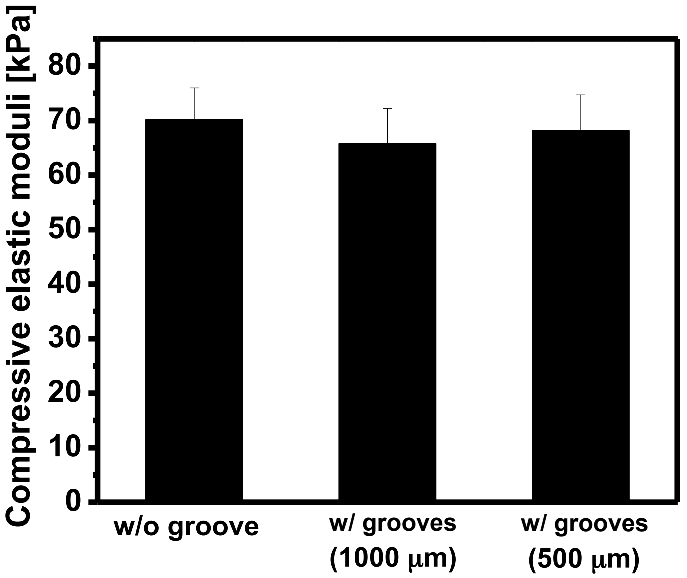

Figure S1. The compressive elastic moduli of flat and micro-grooved alginate hydrogels. The spacing between microgrooves was varied from 500 to 1,000 μm.

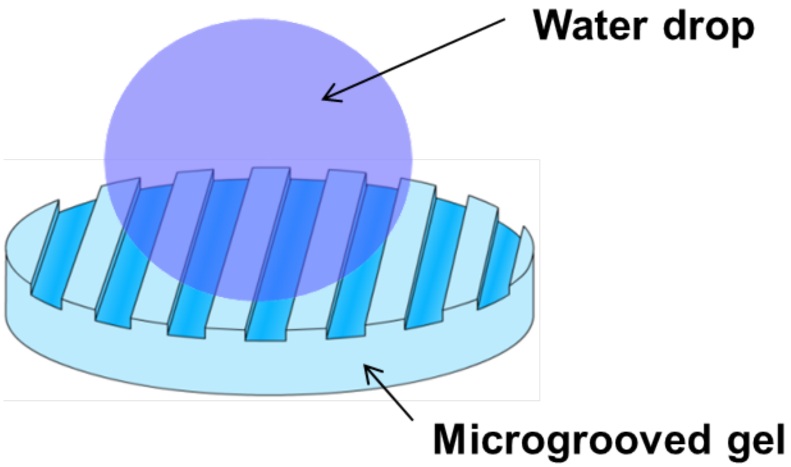

Figure S2. A schematic describing an experiment for the MRI-based monitoring of water diffusion into hydrogels. 50 μl of DI water droplet was placed on top of the 1 cm-diameter gel surface with controlled topologies.

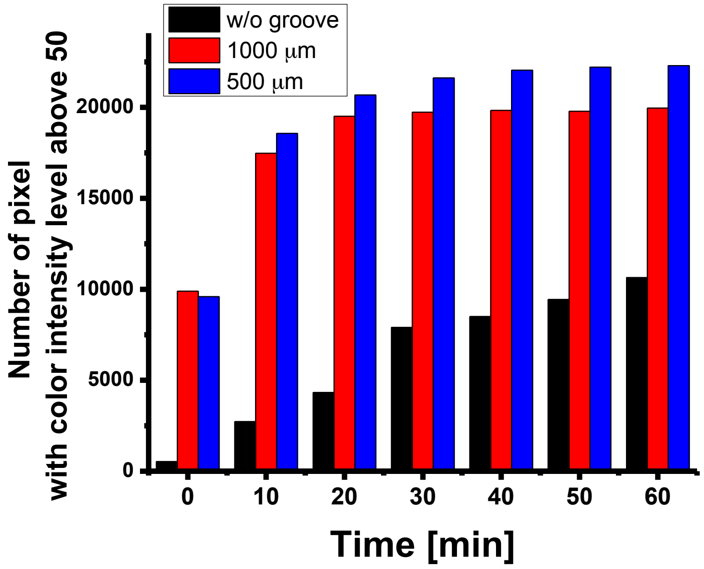

Figure S3. Number of pixels that present positive pseudo-colors with an intensity higher than 50 in MR images of hydrogels in Figure 2C. Black bar represents the hydrogel without microgrooves. The red and blue bars represent the hydrogels with microgrooves of 1,000 and 500 μm-spacing, respectively.

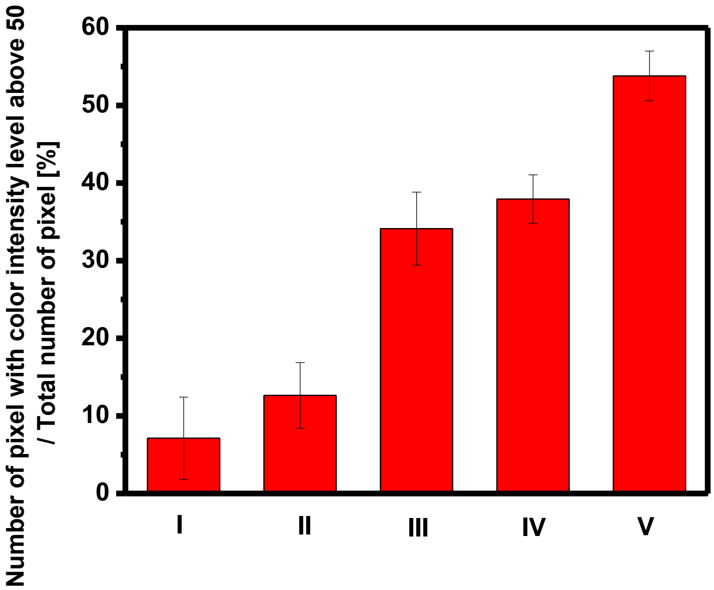

Figure S4. Analysis of the number of pixels that present positive pseudo-colors with an intensity above 50 from MR images of the hydrogels in Figure 3C (I-V). I: microporous gel W/O microgrooves; II: microchanneled hydrogel W/O microgrooves; III: microporous gel with microgrooves; IV: (//) microchanneled gel; V: (⊥) microchanneled gel.

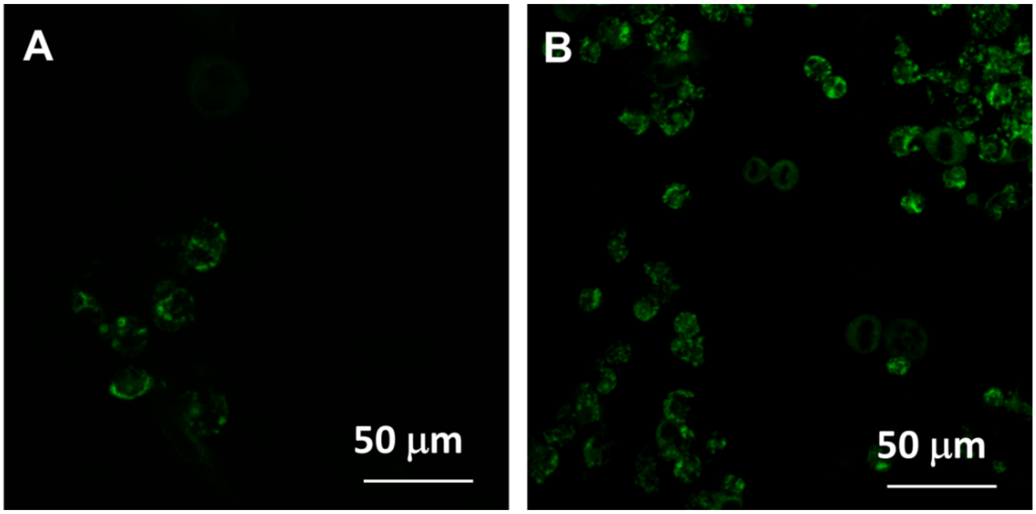

Figure S5. Calcium imaging of undifferentiated (A) and differentiated (B) BMSCs cultured on a glass substrate.

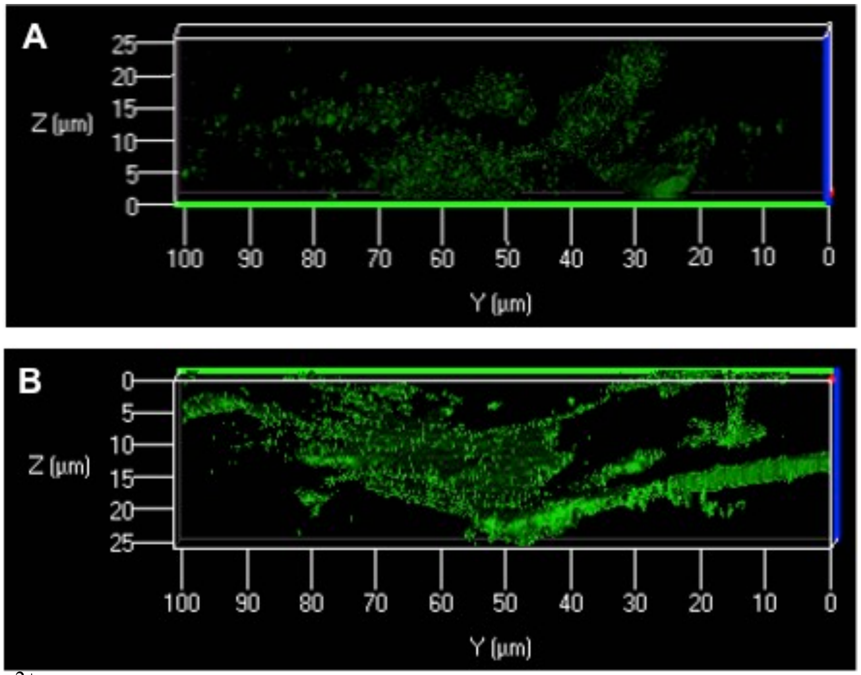

Figure S6. Ca^{2+} images of differentiated neurons within a microchanneled gel without microgrooves (A) and with grooves of 500 mm-spacing (B). The middle z-axis level corresponds to the depth of 60 micrometer from top surface of the gel.

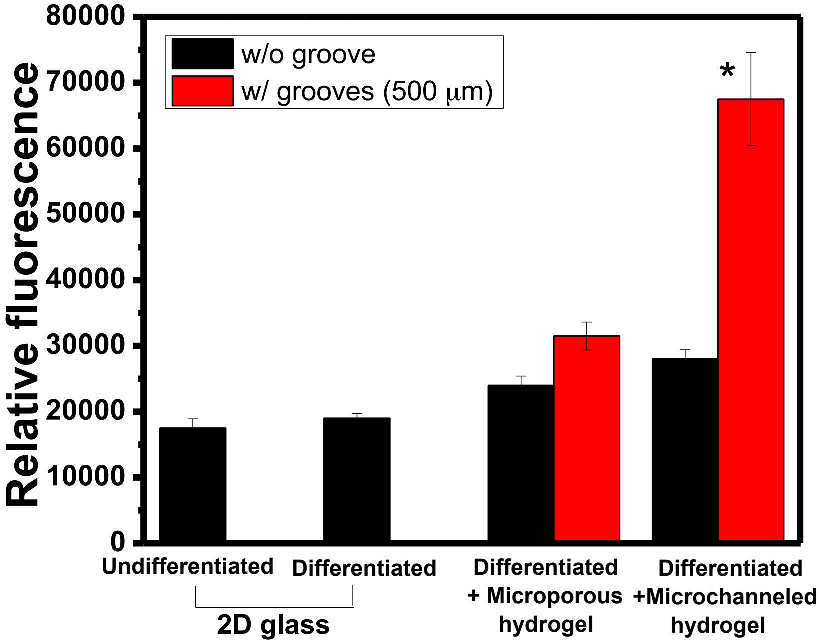

Figure S7. Quantitative analysis of the relative fluorescence obtained from calcium images of the samples.
